# Information Needs of Breast Cancer Patients: Theory-Generating Meta-Synthesis

**DOI:** 10.2196/17907

**Published:** 2020-07-28

**Authors:** Hongru Lu, Juan Xie, Lynette Hammond Gerido, Ying Cheng, Ya Chen, Lizhu Sun

**Affiliations:** 1 School of Information Management Nanjing University Nanjing China; 2 School of Information Florida State University Tallahassee, FL United States; 3 Department of Oncology The Affiliated Shuyang Hospital of Xuzhou Medical University Shuyang China

**Keywords:** breast cancer patients, information needs, incentives, moderating variables, meta-synthesis

## Abstract

**Background:**

Breast cancer has become one of the most frequently diagnosed carcinomas and the leading cause of cancer deaths. The substantial growth in the number of breast cancer patients has put great pressure on health services. Meanwhile, the information patients need has increased and become more complicated. Therefore, a comprehensive and in-depth understanding of their information needs is urgently needed to improve the quality of health care. However, previous studies related to the information needs of breast cancer patients have focused on different perspectives and have only contributed to individual results. A systematic review and synthesis of breast cancer patients’ information needs is critical.

**Objective:**

This paper aims to systematically identify, evaluate, and synthesize existing primary qualitative research on the information needs of breast cancer patients.

**Methods:**

Web of Science, EBSCO, Scopus, ProQuest, PubMed, PsycINFO, The Cochrane Library, the Cumulative Index to Nursing and Allied Health Literature were searched on February 12 and July 9, 2019, to collect relevant studies. A Google Scholar search, interpersonal network recommendations, and reference chaining were also conducted. Eligible studies included qualitative or mixed-methods studies focusing on the information needs (across the cancer continuum) of breast cancer patients or their social networks. Subsequently, a Critical Appraisals Skills Programme checklist was used to assess the quality of included research. The results, findings, and discussions were extracted. Data analysis was guided by the theory-generating meta-synthesis and grounded theory approach.

**Results:**

Three themes, 19 categories, and 55 concepts emerged: (1) incentives (physical abnormality, inquiry from others, subjective norm, and problems during appointments); (2) types of information needs (prevention, etiology, diagnosis, clinical manifestation, treatment, prognosis, impact and resumption of normal life, scientific research, and social assistance); (3) moderating variables (attitudes, health literacy, demographic characteristics, disease status, as well as political and cultural environment). The studies revealed that the information needs of breast cancer patients were triggered by different incentives. Subsequently, the patients sought a variety of information among different stages of the cancer journey. Five types of variables were also found to moderate the formation of information needs.

**Conclusions:**

This study contributes to a thorough model of information needs among breast cancer patients and provides practical suggestions for health and information professionals.

## Introduction

Breast cancer is one of the most commonly diagnosed malignant tumors worldwide [1]. In 2018, around 2.1 million new female breast cancer cases were diagnosed worldwide, accounting for almost 25% of the cancer cases among women [1]. In the United States, an estimated 42,260 breast cancer deaths were predicted in 2019 [2]. The dramatic growth in the number of breast cancer patients has led to an increasing need for information to manage symptoms [3], make decisions, control lives, and prepare for the future [4]. However, information needs have not been thoroughly examined. For example, a lack of targeted education materials has been reported in clinical practice [3]. Several large-scale patient investigations have identified inadequate and inaccessible patient information [5]. Therefore, it is crucial and challenging for health service providers to systematically investigate patients’ information needs and offer relevant information.

We identify two existing reviews on breast cancer patients’ information needs. A review of relevant literature published between 1988 and 1998 classified individual studies to examine information needs and source preferences of breast cancer patients and their family members at different points in their cancer journey [6]. They found that essential information needs changed over time, and family members also needed information to support the patients. A recent protocol for a scoping review has been put forward that aims to summarize studies exploring information needs, source preferences, and engagement behaviors of women with a specific breast cancer type (metastatic) [5]. However, it does not address the specific information needs of general breast cancer patients, and no further findings have been published. Therefore, there is no updated review and synthesis of breast cancer patients’ information needs.

There is a large body of qualitative studies that have mainly collected data through interviewing and nonparticipatory observation on this topic. These studies contribute to an insightful understanding of patients’ information needs and characteristics [7], but they have mostly focused on particular topics with limited samples and produced mixed results. For example, different results have identified the information needs of patients in specific age groups [8,9]. Therefore, a qualitative meta-synthesis will help to address the mixed results and establish a general model of the information needs of breast cancer patients.

To narrow these gaps, this study aims to identify, evaluate, and synthesize existing primary qualitative research on the information needs of breast cancer patients and generate an integrated model to articulate their information needs (ie, incentives, types and moderating variables) across the cancer care continuum. The findings can inform health professionals and information service providers to help breast cancer patients receive appropriate information and be well equipped to cope with the disease.

## Methods

This study follows the processes of theory-generating meta-synthesis [10] and grounded theory [11].

### Search Strategy

The search strategy was first developed for Web of Science (Clarivate Analytics) and then adjusted to search EBSCO, Scopus, ProQuest, PubMed, PsycINFO, The Cochrane Library, and the Cumulative Index to Nursing and Allied Health Literature. These databases were chosen based on related studies [12,13]. In addition, a Google Scholar search, interpersonal network recommendations, and reference chaining of the included articles were applied. The literature search was conducted on February 12, 2019, and updated on July 9, 2019. Duplicate articles were removed.

Search terms were chosen from two categories: breast cancer patients and information needs. The search terms were chosen according to MeSH vocabulary [14] and related research on information behavior [15-20] (see Multimedia Appendix 1 for the detailed search strategies). “Behavio*,” “seek*,” “source*,” and other words were added so all related studies could be identified since findings on information needs may have also been covered in information behavior research (eg, what information did the patients search?). Limits on research methods were not initially placed on the search terms since it was difficult to accurately identify all qualitative studies when searching for the literature on this topic in the databases [21]. Therefore, qualitative studies were selected in the screening process.

### Selection Criteria

Articles were selected according to inclusion and exclusion criteria [12,13] (Textbox 1).

**Selection criteria for the study.**

Inclusion criteria:

- Empirical studies using qualitative or mixed research methods
- Studies focusing on breast cancer patients or support groups encompassing breast cancer patients (can include other types of patients)
- Studies related to information needs

Exclusion criteria:

- Reviews or patents
- Mixed-method studies from which no qualitative findings can be extracted
- Studies focusing on participants with the risk of breast cancer (family history)
- Studies concerning breast cancer patients’ relatives, friends, or spouses
- Studies concentrating on doctors of breast cancer patients
- Studies related to breast cancer patients and other kinds of participants that fail to distinguish breast cancer patients from other groups

### Screening

**Figure 1:**
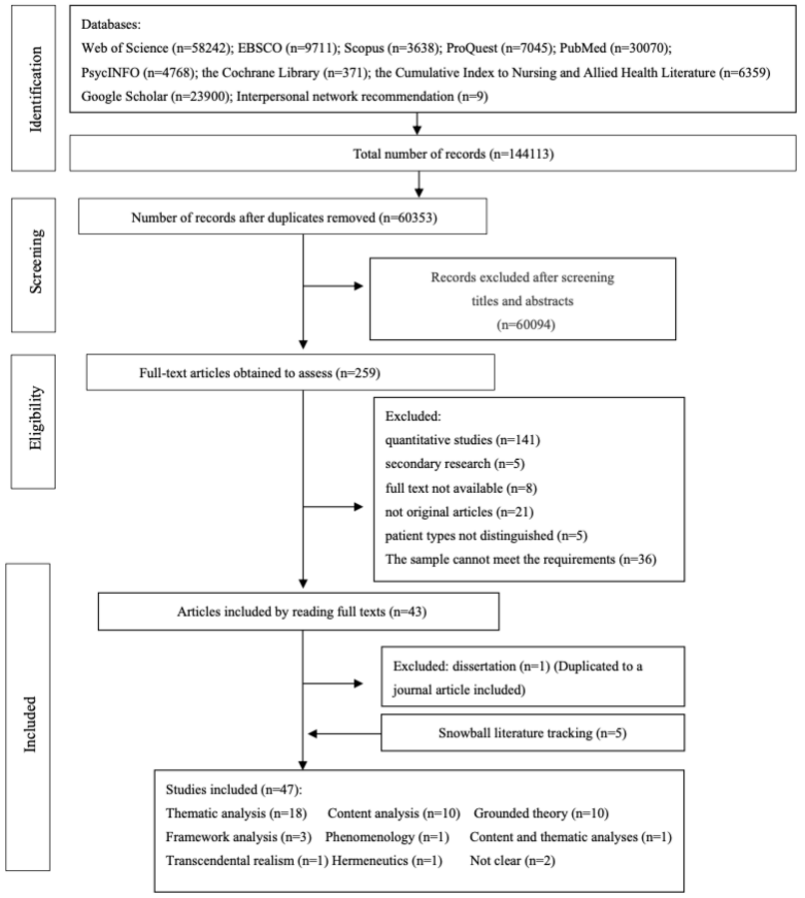
Flowchart of searched, excluded, and included items.

The first author and three members of the research team (Juan Xie, Ying Cheng, and Ya Chen) browsed the titles and abstracts to identify possible studies. The full texts of these studies were then retrieved for further screening based on the inclusion and exclusion criteria. The screening was conducted separately by the four authors, and disagreements were addressed through discussion. An additional 5 articles were found through a snowballing literature track. Finally, 47 full-text articles were included after removing one duplicate. The literature search process is illustrated in Figure 1.

### Quality Appraisal

The quality of the included studies was appraised using the Critical Appraisals Skills Programme [22] (see Multimedia Appendix 2 for scores). As shown, all 47 studies received a score indicating high quality (ie, over 8). Therefore, no paper was excluded through this appraisal. A total of 38 articles received a score of 10, which indicated that they fully reported their research objectives, qualitative research methods, research design, recruitment strategy, data collection methods, relationship between researchers and participants, ethical issues, data analysis, statement of findings, and value of the research [22]. Nine articles scored 9 indicating that they did not clearly describe the research objectives, method of data analysis, or ethics statement.

### Data Extraction and Synthesis

All six researchers independently read and extracted the results, findings, and discussions of the included qualitative studies. To extract as many relevant findings as possible, researchers discussed, reached consensus, and adopted the same codes to analyze the articles.

The extracted data were iteratively synthesized in three stages. First, four authors (HL, JX, Y Cheng, and LS) identified the concepts that best fit the extracted raw texts. Categories were then formed by gathering concepts with the same properties and dimensions through continuous comparison. Second, the relationships among categories were also examined. Themes emerged after an in-depth analysis and classification of categories. Third, concerning the evidence found in the extracted data, all themes were integrated according to their logical relationship. Finally, a model clarifying the information needs of breast cancer patients was built.

## Results

### Overview of the Literature

In all, 47 journal articles were included. The characteristics of the included studies are shown in Multimedia Appendix 2. The data collection methods used in these studies were semistructured interviews, focus groups, observations, an online survey, and/or an open-ended questionnaire. Most studies applied thematic analysis, content analysis, and grounded theory to analyze the data. The recruitment strategies were generally convenience sampling and purposive sampling. These studies were conducted in the United States (13), Australia (12), United Kingdom (7), Canada (10), Turkey (1), Japan (1), Iran (1), Poland (1), and Switzerland (1). As for the populations, 26 studies reported the race of the participants, among which 18 studies focused mainly on white participants (over 60%), and 6 studies recruited all Asian participants (eg, Japanese and Chinese-Australian). Participants of one study were all African American. In addition, one study recruited mixed participants such as Asian, white, and Filipino. In terms of the types of information patients sought, the studies revealed that participants wanted information from various sources, encompassing information from health care providers (47); peers/support teams (24); the internet (18); books (14); cancer organizations (13); families (13); friends/coworkers (13); brochures and pamphlets (12); magazines (9); audio/videos (7); complementary, alternative, or unconventional practitioners (6); newspapers (4); libraries (3); television (3); telephone hotlines (3); medical records (2); and broadcasts (1). In addition, 10 studies used theoretical frameworks, such as planned behavior theory and social learning theory, in their research.

### Synthesized Findings

#### Summary

**Figure 2:**
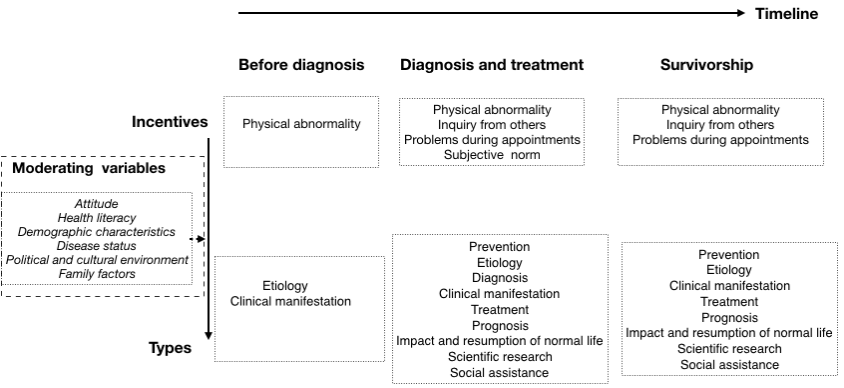
Model of information needs of breast cancer patients.

Three themes emerged to articulate the developing process of information needs of breast cancer patients, with 19 categories and 55 concepts underpinning the themes (Table 1). This study also explored the logical relations between themes and built an integration model (Figure 2) guided by incentive theory [23]. According to the theory, individual activities are not only derived from internal motivation but are also stimulated by external factors, including positive and negative incentives [23]. The process of diagnosis and treatment in breast cancer patients’ journeys is sequential [24] and often accompanied by various diagnostic approaches and treatment regimens in a clinical context [24]. Therefore, diagnosis and treatment were synthesized as one stage in this study (phase 2). Consequently, we identified another two phases. Phase 1 is the stage before diagnosis: before going to the hospital for medical advice or examination in allusion to physical abnormalities. Phase 3 is conceptualized as survivorship. Incentives and content of patients’ information needs vary in each of the phases. In particular, 7 studies reported the content and incentives of information needs before diagnosis (phase 1), while 44 studies focused on diagnosis and treatment (phase 2) and 29 studies presented information needs during survivorship (phase 3). Variables moderating the formation of information needs are also shown in the model.

**Table 1:** Synthesized results of the studies.

Themes, categories, and concepts			Examples of quotations	References
**Incentives**				
	Physical abnormality	Appearance of symptoms of the primary tumor; appearance of symptoms of side effects	“Information needs also became more prevalent when patients experienced side effects...” [25].	[9,25-38]
	Inquiry from others	Inquiry from doctors; inquiry from patients around them; inquiry from general people around them	“I was being asked so many questions from those around me, and I wished if I have asked the physicians these questions and knew its answers” [36].	[26,31,32,36,39]
	Subjective norm	Advice from support group member	“Leigh followed the advice of another medically savvy support group member and requested copies of ‘everything’ regarding her diagnosis” [40].	[40]
	Problems during appointments	Time span; perfunctory doctors; changes in health care staff; inconsistent information	“When this time frame was more than 4 weeks, patients found it hard to remember everything that had been discussed. They therefore had additional information needs that required attention during their planning appointment” [25].	[25,30,37,41]
**Types of information needs**				
	Prevention	Effectiveness of breast self-examination; prevention for family members	“Themes related to important content issues include:...prevention for daughters” [30].	[30,40,42,43]
	Etiology	Internal factors; external factors	“The exchange of misinformation also led many of the women interviewed...to hold misconceptions about breast cancer including misconceptions about risk factors...of the disease” [36].	[36,40,42,44]
	Diagnosis	Specific biopsy procedures; pathologic results; precision and applicability of examination tools; explanations of technical terms related to diagnosis; clinical stage; waiting time of diagnostic tests and its impact on prognosis	“Faced with having to go for a mammogram, the women were concerned that this imaging tool lacked the precision needed to detect a tumor through dense breast tissue...the mammograms don’t work with young women” [9].	[9,36,40,43,45-47]
	Clinical manifestation	Symptoms of the primary tumor; symptoms of side effects; meaning of corresponding symptoms	“Participants reported a preference for a list of signs and symptoms of breast cancer recurrence as a means to reduce unnecessary anxiety” [48].	[30,33,36,37,43-45, 48-50]
	Treatment	Treatment options; side effects of treatment; management of side effects; treatment preparation; treatment procedures; treatment evaluation summaries; prevention of recurrence	“A small number of women also reported asking the health care provider which treatment option they would recommend” [28].	[8,9,25,26,28-66]
	Prognosis	Survival rates; risk of complications; survival statistics of treatment regimens; risk of recurrence	“The search for survival statistics proved fruitless, though, since such information cannot simply be applied to an individual case to determine prognosis” [26].	[26,27,30-32,42,46,47, 51,54,55,59,66]
	Impact and resumption of normal life	Ways to communicate the diagnosis results with family members; impact of treatment on quality of life; strategies to improve quality of life	“Patients had information needs relating to...whether they can return to work and other health services” [25].	[8,9,25-30,32-36,38, 39,41-44,47,48,51-53, 56,60-64,67-69]
	Scientific research	Recent research findings; clinical trials	“...every week there’s another breakthrough. And you go to your doctor with the clipping” [30].	[30,40,44,47,65]
	Social assistance	Insurance; financial support	“Women’s careers were often affected by the illness yet information about ﬁnancial support was extremely difﬁcult to come by” [52].	[9,26,30,43,46,52,60]
**Moderating variables**				
	Attitudes	Affect; behavior; cognition	“Some women talked of being shocked, frightened, and worried when discovering a breast lump and immediately sought medical advice. Other women appeared less concerned and mentioned breast symptoms only when attending their general practitioner for other reasons” [28].	[8,9,25,26,28,33,35, 36,46,48,57]
	Health literacy	Health beliefs; health knowledge; health styles	“The possibility of symptoms being breast cancer was the first thought of some women and most were fatalistic about this. ‘I just thought I have cancer and I wasn’t bothered about it because let sleeping dogs lie. The less you know the less you bother about it’” [28].	[9,26,28,33,34,40]
	Demographic characteristics	Age; education level; economic status	“Ongoing information needs...younger women also discussed the use of complementary therapies...more than their older counterparts” [44].	[8,25,28,40,42,44,57,64]
	Disease status	Comorbidity; clinical stage	“Experiences of some participants with special disease showed they needed information regarding secondary prevention” [42].	[41,42,60]
	Political and cultural environment	Cultural background; health care policy	“Given that food therapy plays a significant role in Chinese culture, many participants expressed a strong desire for information on diet” [49].	[30,32,33,35,38,49]
	Family factors	Age of children	“At diagnosis, women wanted age-appropriate information about how to communicate with their children about cancer” [9].	[9]

#### Theme 1. Incentives

This theme illustrates how breast cancer patients formed a cognitive state of information gaps, which led to their information needs. These needs were triggered by various factors.

##### Physical Abnormalities

Breast cancer patients might experience physical abnormalities anytime during the cancer continuum. Their information needs were often triggered by the appearance of symptoms of the primary tumor (eg, lumps, pain, or discharge) [9,26,28,31-33,36] because patients’ panic and uncertainty increased before diagnosis. Nine studies also reported side effects caused by the treatment (eg, irregular menstruation, arm complications, fatigue, sexual dysfunction, lymphedema, and pain) [25,27,29,30,32,34,35,37,38] that would last from the treatment to the survivorship stages and could lead to specific information needs.

##### Inquiry From Others

Three studies suggested that doctors provided patients with several surgery choices and asked patients to quickly make treatment decisions (in phase 2), resulting in information needs about treatment [26,32,39]. During treatment (in phase 2), a patient wished that she had asked doctors more questions because she could not answer the queries of patients around her, indicating that other patients’ questions helped form information needs that some patients themselves were not aware of [36]. In the survivorship stage, a patient was asked questions by general people around her, which reminded her of the risk of a recurrence, leading her to go back to the clinic for a follow-up appointment [31].

##### Subjective Norm

Patients’ information needs were also triggered by subjective norms (“the belief that an important person or group of people will approve and support a particular behavior” [70]). For example, in phase 2, one patient reportedly requested copies of all her diagnostic results from the hospital [40], following the advice from a support group member.

##### Problems During Appointments

Similar to the inquiries from others, problems could occur during appointments. For example, during the process of treatment decision making, the long waiting period between diagnosis and making a final treatment decision made it difficult for patients to remember all the information discussed, thus generating more information needs [25]. In addition, the doctors sometimes did not provide patients with enough information about breast cancer treatment options and simply made the treatment decision without discussion with the patient, which left the patient with more uncertainty [41]. The patients also had many questions (eg, who and how often should they see the doctor) as they transitioned from the cancer care group to the primary care physician shortly after treatment (in phase 3) [30,37]. Patients were also confused when health care professionals provided them with inconsistent information (eg, tamoxifen discontinuation) in the survivorship stage [30], triggering the need for more exact information. In a follow-up appointment (in phase 3), it was reported that the doctor’s indifferent attitude also led to patients’ anxiety and uncertainty when doing a routine examination [30].

#### Theme 2. Information Needs

This theme refers to specific information patients needed. The included studies suggested that breast cancer patients generally had 9 types of information needs at different stages of cancer care including information on prevention, etiology, diagnosis, clinical manifestation, treatment, prognosis, impact on normal life and coping, research progress, and social assistance. The types of information needs can be divided into several concepts.

##### Prevention

Three studies reported that patients were concerned about information on prevention, including effectiveness of breast self-examination [40] in phase 2. Patients reported that they also needed information about prevention for family members [30,42,43] during the survivorship stage.

##### Etiology

Etiology information was needed throughout the whole cancer journey. Four studies reflected on this type of information need. For example, patients wanted to know about internal and external factors (risk factors) before diagnosis in phase 1 [36] and phase 2 [40]. Patients also needed information on internal (genetic) [42,44] and external factors (possible carcinogens and environmental triggers) [44] in the survivorship stage in phase 3.

##### Diagnosis

In the diagnosis and treatment phase (in phase 2), patients were concerned about specific biopsy procedures (arrangement, temporal sequence, and actual procedure) [45], pathologic results (results of the biopsy) [45], and precision and applicability of the examination tools [9]. They also wanted an explanation of technical terms related to the diagnosis (used to describe tumor types) [46], pathologic results (pathological stage or the type of breast cancer) [36,43,46], clinical stage [36,40], waiting time of diagnostic tests, and the corresponding impact on prognosis [47].

##### Clinical Manifestation

Patients wanted to know about the signs of breast cancer (symptoms of the primary tumor) [36] before diagnosis (phase 1). Information about the meaning of the corresponding symptoms was needed by patients who were undergoing a breast biopsy (in phase 2) [45]. They also needed to be informed of the symptoms of the side effects caused by the treatment [30,37] during phases 2 and 3. Eight studies also reported that patients were concerned about symptoms of a recurrence (symptoms of the primary tumor) [30,33,37,43,44,48-50] during phase 3.

##### Treatment

Patients needing treatment information during the cancer continuum was commonly reported across studies (n=44): 23 studies found that patients needed treatment options while waiting for diagnosis results [45], shortly after receiving diagnosis results [36,40,51], and during the treatment decision process [8,9,26,28,31,32,34,39,40,42,46-48,52-59] such as popularity, advantages and disadvantages, risks, applicability, recovery time, comparison, cost, and optimal time of different treatment options in phase 2. Twelve articles also mentioned that patients wanted to be educated about the side effects of treatment [8,9,25,28,31,36,40,46,56,60] when considering the treatment decision. The information needs of patients during treatment preparation included details about treatment preparation [33,42,48,52] and treatment procedures [9,25,28,30,42,46,61,62].

During treatment, patients’ information needs focused on the side effects of treatment [25,47] and management of the side effects [8,25,38,40,46,49,50,53,60,63,64]. The studies also reported that patients needed details on the treatment options (alternative and complementary medicines and treatment) [25,65] and treatment procedures (eg, location of the pharmacy) during treatment [28,40,42,46,53,57,61]. In the survivorship stage, patients also paid attention to information on side effects [8,29,30,32,33,36-38,41,42,48] and management of side effects [25,30,35,37,43,48,49]. In phase 3, patients needed information about treatment options (eg, complementary and alternative therapies) [42,44,64,65] and treatment evaluation summaries [43,48]. In addition, patients wanted information about preventing a recurrence (eg, prevention-related policies, signs and symptoms of a recurrence, natural remedies to prevent recurrence, preventive health actions to minimize the risk of a recurrence) [30,33,37,41-43,49,52,61,66] in the survivorship stage.

##### Prognosis

Patients’ need for prognosis information included general survival rates [26,47,51,54] and the risk of a recurrence [27] in phase 2. Studies also found that patients desired information about the risk of complications [42], risk of a recurrence [31,46], and survival statistics of treatment regimens [55,59] when they made treatment decisions. In particular, patients needed to know the risk of a recurrence in phases 2 [47] and 3 [30,32,66].

##### Impact and Resumption of Normal Life

Patients wanted to know how to communicate about the cancer with their family members in phase 2 [9,26,52,67,68]. Patients also needed information about the impact of treatment on their own body image [28,32,33,35,42,62,69], fertility (or menopause) [8,35], work [25,26], and psychology [8,26,64] in phase 2. In the survivorship stage, patients were concerned about the impact of treatment on work [44,52], intimate relationships [44,69], fertility (or menopause) [9,30,44], psychology [42,44,69], and body image [9,27,34,35,69]. In 26 studies, strategies to improve patients’ quality of life (eg, fertility preservation options, sex advice, instructions about diet and exercise, stress management, reconstruction) were important for patients in both phase 2 [9,26,27,35,38,39,47,51,53,56,60-62] and phase 3 [29,30,32,36,41-44,48,63,64,69].

##### Scientific Research

Some patients needed information about recent breast cancer research findings [40] and clinical trials [47,65] in phase 2. In addition, medical breakthroughs (recent research findings), corresponding clinical trials [30], new treatment, and research developments [44] were requested in phase 3.

##### Social Assistance

Patients generally needed information about insurance (eg, health insurance) in phases 2 [46] and 3 [30]. Five studies reported that patients sought information on financial support (eg, employment benefits, where to get help) during the survivorship stage [9,26,43,52,60].

#### Theme 3. Moderating Variables

A prominent theme reported in the included studies was variables that moderated the development of information needs of breast cancer patients. Differences among particular subgroups and their forms of information needs are shown in Table 2. No major inconsistencies were detected in the studies.

**Table 2:** Synthesized results of the moderating variables.

Categories, concepts, and dimension			Effect on information needs	References
**Attitudes**				
	**Cognition**			
		Correct cognition	Facilitated development of information needs	[9,26,28]
		Misunderstanding	Inhibited development of information needs	[33,36]
	**Affect**			
		Shocked and worried	Facilitated development of information needs	[28,57]
		Not worried	Inhibited development of information needs	[28]
	**Behavior**			
		Ready	Facilitated development of information needs	[8,35,46,48]
		Not ready	Inhibited development of information needs	[25]
**Health literacy**				
	**Health knowledge**			
		Rich in health knowledge	Facilitated development of information needs	[33,40]
		Lacking health knowledge	Inhibited development of information needs	[33]
	**Health style**			
		Positive coping strategies	Facilitated development of information needs	[33]
		Negative coping strategies	Inhibited development of information needs	[34]
	**Health beliefs**			
		No fatalism	Facilitated development of information needs	[9,26,34]
		Fatalism	Inhibited development of information needs	[28,33]
**Demographic characteristics**				
	**Age**			
		Younger	Paid more attention to information on new treatments, research advances, and effects of treatment on fertility and career, complementary therapies, dietary changes and exercise, possible carcinogens, and environmental factors	[8,25,44]
		Older	Expressed less need for reconstructive surgery	[28,40,42]
	**Education level**			
		Higher	Paid more attention to information on medical terminology and medical information systems	[40]
		Lower	Inhibited the development of information needs	[40]
	**Economic status**			
		Higher	Paid more attention to information on natural health products and healthy dietary changes	[57,64]
		Lower	Expressed less need for healthy diet information and reconstructive surgery	[42]
**Disease status**				
	**Comorbidity**			
		With comorbidity	Paid more attention to information on secondary prevention; obese patients needed survival guidelines targeting their physical condition	[41,42]
	**Clinical stage**			
		Advanced breast cancer	Paid more attention to the experience of other advanced breast patients; information or support related to last will and testament and final arrangements	[60]
**Political and cultural environment**				
	**Cultural background**			
		Chinese	Paid more attention to diet and exercise guidelines, less information on postoperative body changes	[33,38,49]
		Turkish	Paid more attention to postoperative body changes and contraceptive information	[35]
		Japanese	Paid more attention to information on postoperative body changes	[32]
	**Health care policy**			
		Policy changes	Paid more attention to information on changes in health care policy and practice (eg, frequency of routine examination)	[30]
**Family factors**				
	**Age of children**			
		Younger	Paid more attention to age-appropriate information on how to guide communication with children about the disease	[9]

##### Attitudes

Attitudes refer to the amount of affection for or against some object [71]. In this study, attitudes included affect, behavior, and cognition. Affect represented the strong feelings of breast cancer patients, such as being shocked and worried [28,57], while behavior described their readiness to receive information [25]. Cognition was the product of knowledge acquisition or application, such as an understanding of breast cancer.

Six studies showed that patients’ cognition and affect influenced the development of information needs. For example, some patients misunderstood the causes and risks of breast cancer, believing that they would not have breast cancer after a certain age [33] or after breastfeeding [36], and thus they did not seek information. Correct cognition of patients facilitated the development of their need for information. For example, patients went to see their physicians after discovering a lump or feeling pain [9,26,28]. Two studies showed that affect also influenced their information needs. For instance, some participants who found a lump and felt shocked immediately went to the hospital to seek medical advice [28,57], while those who were not worried about the lump only described the symptoms when they went to see their general practitioner for other diseases or they chose to completely ignore the lump [28].

In addition, some patients who believed that they were still in the process of receiving diagnosis results were not willing to process a large amount of information [25]. Patients who lacked behavior intention chose to avoid information [25], while other patients asked for various types of information [8,35,46,48].

##### Health Literacy

Health literacy is defined as individuals’ ability to obtain, understand, evaluate, and apply health information, which can affect individual judgments and decisions on health care and disease prevention [72]. In the study, this category included health beliefs, health knowledge, and health styles, respectively, representing patients’ values toward breast cancer, awareness of cancer-related information in order to seek appropriate health services [73], and their coping strategies for breast cancer.

For example, one patient did not go to see a doctor after discovering a lump in her breast because she lacked health knowledge about the effect of screening tools (mistakenly believing that routine mammographic screening had a preventive effect), which inhibited the development of her information needs [33]. Relatively abundant health knowledge could promote the articulation of the need for information [33,40]. While one women who had a different health style also ignored the discovery of lump until she found that the lump was getting bigger 3 month later, other patients went to see a doctor immediately after feeling breast pain [33]. In terms of health beliefs, some of the patients suspected breast cancer immediately after finding the lump, but they were not worried or did not ask about it because of fatalism [28,33] whereas other patients sought further examination [9,26,34].

##### Demographic Characteristics

Eight studies reported that the patients’ age, education level, and financial situation moderated the formation process of their information needs. For example, young patients cared more about new treatments, research advances, and effects of treatment on fertility and career compared to older patients [8,25,44]. Younger patients also expressed a greater need for information about complementary therapies, dietary changes and exercise, possible carcinogens, and environmental factors of breast cancer because of their interest or a sense of control and comfort [44]. In contrast, some older patients had less need for information and considered that the less they knew, the less they were bothered about it [28]. In general, patients with a lower education level were more likely to trust doctors and be satisfied with the information provided by their doctors [40]. These patients believed that they did not need more information and had fewer information needs than other groups [40]. In terms of education levels, having a high education level had a beneficial effect on information needs and they independently researched areas such as medical terminology and medical information systems [40]. Furthermore, patients with lower economic status showed no concern about healthy diet information due to the high costs as well as the conflicts between a special diet and their family’s diet [42]. In contrast, others cared about natural health products and healthy dietary changes [57,64]. It was also found that worse economic status and older age inhibited their need for information on reconstructive surgery [42].

##### Disease Status

Disease status involved comorbidity and the clinical stage of patients. For patients with comorbidity, information on secondary prevention was needed to avoid complications [42]. For example, obese patients faced a higher risk of recurrence, and they needed targeted survival guidelines based on their physical condition [41]. Patients with advanced breast cancer also wanted to know about the experiences of other patients in the same clinical stage [60]. They also sought information or support related to the last will and testament and final arrangements [60].

##### Political and Cultural Environment

The political and cultural environment refers to the social and cultural context characterized by a community’s values and beliefs [74]. Of the included studies, 6 suggested a moderating effect of cultural background and health care policy. Patients who were influenced by Chinese culture developed more information needs regarding specific recommendations on diet and exercise related to symptom management, promotion of rehabilitation, and prevention of recurrence [38,49] because of the traditional food therapy culture. Some Chinese patients reported no feeling about postoperative body changes because body image was not as important to them as returning home to take care of their families in the context of Chinese culture [33]. In contrast, patients in some other countries like Turkey [35] and Japan [32] attached great importance to the aesthetic needs of the female body image. In addition, for patients from some countries with religious beliefs like Turkey with strong opposition to abortion, the cultural beliefs directly affected the patients’ need for contraceptive information [35]. Patients in countries that had experienced changes in the national health care policy (eg, national cost curtailment policy) and corresponding adjustments in practice (eg, frequency of routine examinations) needed more targeted information on these changes [30].

##### Family Factors

In addition to the moderating variables described above, one study found that patients who had younger children often talked about the lack of personalized information for their kids [9]. They particularly needed targeted information on how to guide their communication about breast cancer with their children because they had difficulty finding age-appropriate information [9].

## Discussion

### Principal Findings

This meta-synthesis strengthens our understanding of the formation and types of information needs of breast cancer patients. It also highlights the variables moderating the development of information needs.

#### Information Service Targeted on General Breast Cancer Patients

Figure 2 shows that breast cancer patients’ information needs change over the three stages. Patients are often given substantial resources (eg, brochures, books, and nursing plans) but the information has disadvantages in both content and form. Targeted messages are often embedded in a large amount of irrelevant information. Breast cancer patients are also often given overwhelming information during hospitalization, putting extra pressure on them [75]. In addition, the information is not always delivered in expected, easy-to-digest forms, but is often filled with medical jargon, for instance [3]. This study provides some practical recommendations based on synthesized findings to guide medical professionals attempting to provide better information for breast cancer patients.

In this synthesis, breast cancer patients’ information needs were systematically examined and classified into three cancer phases. This can help us understand the changing process of the specific information needed by patients at different phases. The synthesized findings also inform recommendations for health care staff to offer relevant and timely education materials at the appropriate stage of the cancer journey. A total of 94% (44/47) of the studies reported that patients were concerned about treatment information such as treatment procedures, side effects, and preoperative preparation. This type of universal information should be incorporated into education materials to help patients make proper treatment decisions. The synthesis also revealed that it is difficult for patients to obtain information about financial assistance [9,26,30,52,60], as well as complementary and alternative therapies [34,64]. More attention should be given to meet these information needs.

Easy-to-understand materials are also recommended since much the existing information and oral communication seems to contain complex medical language [76]. Education programs using virtual reality technology could also increase the knowledge and positive experience of patients [77]. Graphics and tables can also help patients understand the cancer care information.

#### Information Services Targeting Specific Groups of Breast Cancer Patients

##### Attitudes of Patients

The synthesis revealed that patients’ attitudes greatly affected their information needs [9,25,28,46]. Numerous other studies have also verified this view. For instance, individual cultural beliefs, views on health and disease, and other factors could affect women’s dealing of early diagnosis [78,79], which is an essential strategy for preventing breast cancer [80,81]. Emotional distress may also affect patients’ ability to cope with the symptoms and treatment of cancer [82].

To date, doctors have not paid enough attention to the emotional pressure patients feel when confronted with a diagnosis and treatment of breast cancer [83]. Thus, social support (eg, support groups, online health communities, and eHealth mobile apps) can play an active role in assisting patients with stress management and providing high-quality care [84,85]. Health care staff can also provide psychological support for breast cancer patients [86] by providing more mental health information. They can also encourage patients to express their genuine emotions and be willing to listen [87]. Finally, it is necessary for medical staff to give full respect to patients and protect their privacy [61].

##### Health Literacy of Patients

This study found that breast cancer patients’ information needs can be influenced by health literacy [28,33,34]. Patients with limited health literacy commonly had more unsatisfied information needs [88]. Health literacy could also influence patients’ cancer screening knowledge, attitudes, and behavior [89]. Inaccurate health beliefs or lack of health knowledge could lead to patients overlooking physical abnormalities, which could affect early detection and treatment of diseases.

People can often detect and treat breast cancer early by improving their health literacy. A higher level of health literacy can also promote effective communication with medical staff and increase patients’ engagement in medical decisions [90]. For patients, limited literacy is associated with a low education level and socioeconomic status [91]. For medical practitioners, the knowledge of prevention, causes, early symptoms, and self-examination methods could be communicated better through mass media to improve patients’ awareness [92]. Furthermore, educational materials on breast cancer prevention should be sensitive to health beliefs [93] to reduce women’s fears of breast cancer screening and fatalism toward the disease. Physicians and other health care providers should continue to give priority to patients’ quality of life when determining treatment plans as patients’ decisions may be influenced by health knowledge. Additional training would help doctors communicate more effectively with patients with low health literacy and reduce medical inequalities.

##### Demographic Characteristics, Disease Status, and Political and Cultural Environment of Patients

The significant increase in the number of breast cancer patients has resulted in diverse and complex information needs. For example, there were more unmet information needs among breast cancer patients who had a foreign native language background (eg, immigrants) [94]. Moreover, the age-specific information needs of younger breast cancer survivors is still far from satisfactory [95]. Therefore, tailored information is recommended to help subgroups of breast cancer patients cope with cancer and improve satisfaction levels [96].

As shown in Table 2, this synthesis reveals suggestions and implications for tailored information services. First, some studies suggested that information provided to younger patients could focus more on the impact of treatment on fertility [8,9,35,44], lifestyle, and career as well as corresponding coping strategies [25,44,52]. More detailed advice on diet could also be provided for patients including many Asian patients. Patients who have young children can also be educated on how to communicate with their children. If patients are adequately informed about body changes before treatment, those who care a lot about body image [32,35] might have more time to consider and prepare for the changes. Policy information should also be given to patients who live in a country with changing medical policies. For patients with comorbidities and other physical conditions, specific prevention and survival guidelines are vital. Since different treatment options have corresponding side effects, health prescriptions that are targeted to each type of treatment can be distributed to patients separately to help them improve symptom management.

### Limitations

Whereas the findings of this study offer significant insights, the limitations can lead to recommendations for further study. Some of the included studies did not clearly report cancer stages, making it difficult to analyze the findings according to the timeline of the disease journey. In addition, this theory-generating meta-synthesis only focused on qualitative studies, which could lead to the omission of valuable findings in quantitative research. Further research is needed to fully understand the unique information needs of breast cancer patients at all stages of their journey.

### Conclusions

This synthesis has identified different information needs at various stages of the cancer continuum. Among the information needs, patients generally pay more attention to information about treatment and prognosis, as well as impact and resumption of normal life. Moderating variables were also identified. The generated model describes a complete pattern of the formation of information needs. Thus, this study contributes to a deeper understanding of breast cancer patients’ information needs and provides practical suggestions for health professionals and information service providers. In particular, health care providers can offer educational materials according to the information needs identified in this study. More personalized information can also be developed to tailor patients’ needs with reference to the moderating variables that influence their information needs.

## Appendix

Multimedia Appendix 1 Search strategies and results.

Multimedia Appendix 2 Included articles and quality assessment results.
