# Supplementary material for: Information Needs of Breast Cancer Patients: Theory-Generating Meta-Synthesis
Source: J Med Internet Res. 2020 Jul 28;22(7):e17907. doi: 10.2196/17907 (PMC7420822; doi:10.2196/17907)
Supplement: Multimedia Appendix 1 [file jmir_v22i7e17907_app1.docx]

| Search date: February 12 and July 9, 2019 | | | |
| --- | --- | --- | --- |
| Database | search terms | Search strategies | Results |
| Web of Science | #1 (“breast cancer*” or “breast tumor*” or “breast neoplas*” or “breast carcinoma*” or “breast oncolog*” or “mammary cancer*” or “mammary tumor*” or “mammary neoplasm*” or “mammary carcinoma*” or “mammary oncolog*”)  #2 (information* or knowledge)  #3 (behavio* or seek* or search* or retriev* or forag* or “look* for” or gather* or collect* or acqui* or need* or requirement* or motivat* or demand* or practice* or use or using or utilis* or utiliz* or “decision mak*” or collaborat* or cooperat* or coordinat* or shar* or select* or evaluat* or assess* or literacy or exchang* or communicat* or process* or manag* or extract* or source* or channel* or avoid* or barrier*) | (Topic=#1) AND (topic=#2) AND (topic=#3) | 58242 |
| Scopus |  | (TITLE-ABS-KEY =#1) AND (TITLE-ABS-KEY =#2) AND (TITLE-ABS-KEY =#3) | 3638 |
| Ebsco |  | (AB=#1) AND (AB=#2) AND (AB=#3) | 9711 |
| proquest |  | (AB=#1) AND (AB=#2) AND (AB=#3) | 7045 |
| PsychoINFO |  | (TX=#1) AND (TX=#2) AND (TX=#3) | 4768 |
| The Cochrane Library |  | (All text=#1) AND (All text =#2) AND (All text =#3) | 371 |
| The Cumulative Index to Nursing and Allied Health Literature |  | (AB=#1) AND (AB=#2) AND (AB=#3) | 6359 |
| Pubmed |  | (All Fields=#1) AND (All Fields=#2) AND (All Fields =#3) | 30070 |
