# Supplementary material for: Information Needs of Breast Cancer Patients: Theory-Generating Meta-Synthesis
Source: J Med Internet Res. 2020 Jul 28;22(7):e17907. doi: 10.2196/17907 (PMC7420822; doi:10.2196/17907)
Supplement: Multimedia Appendix 2 [file jmir_v22i7e17907_app2.docx]

| First author; The year; countries | Sample size | Characteristics of participants | Race of participants | Data collection method | Data analysis method (qualitative) | Sampling method | Research purpose | Theoretical framework | Casp  score |
| --- | --- | --- | --- | --- | --- | --- | --- | --- | --- |
| [1] Banu Karaöz;  2010;  Turkey | 20 | Age; Months since diagnosis; Number of children; Education level; Employment status; Treatment; Cancer stage; Menstrual status; Hormonal adverse effects; Current method of contraception | Unreported | Face-to-face, in-depth,  semi-structured interview | Content analysis | Convenience sampling;  Purposive sampling | Information needed and received by premenopausal Turkish women | None | 10 |
| [2] M. Tsuchiya;  2009; Japan | 12 | Age; Nationality; Marital status; Occupation ; Education level; Treatment; Adjuvant therapy | Japanese | Face-to-face, semi-structured interview | Grounded theory | Convenience sampling;  Theoretical sampling  (Purposive sampling) | Perceived information needs among female BC patients in Japan | None | 10 |
| [3] Ellen Balka; 2010; Canada | 35 | Age; Annual income; Marital status; Employment status; Education level | Unreported | Online survey (allowed respondents to anchor their experiences to specific time points and events) | Grounded theory | Convenience sampling; Purposive sampling | Internet health information-seeking journey undertaken by a specific group of patients | None | 10 |
| [4] Ross E. Gray; 1998; Canada | 70 | Years since diagnosis; Geographical location; Age; Education level; Annual family income; Marital status; Employment status | Unreported | Focus group | Thematic analysis | Convenience sampling; Purposive sampling | Information needs of well, longer-term survivors of breast cancer | None | 10 |
| [5] Jeffrey D.Robinson;  2016; Portland | 132 | Age; Race; Education level; Household income; Marital status; Presence of companion; Formal religious attendance; Days since diagnosis; Cancer stage; Whether first breast cancer; Whether had prior communication with this surgeon | 93.9%White, Non-hispanic and 6.1% other | Non-participatory observation | Grounded theory | Convenience sampling; Purposive sampling | What patients want to know more about prior to treatment | None | 10 |
| [6] Jacqueline Clark; 2005; USA | 35 | Age; Social status; Occupation | Middle-class group were white women; 1/3 of working- class group were women of colors | Field observation; Semi-  structured interview | Grounded theory | Convenience sampling;  Purposive sampling | How class-based inequalities led the women to seek therapeutic information about breast cancer in different ways | None | 9 |
| [7] Rashida Haq; 2013; Canada | 35  (21 breast cancer patients) | Age; Cancer stage at diagnosis; Cancer type; Surgery type; Therapy; Duration of treatment | Unreported | Focus group; individual interview | Thematic analysis | Convenience sampling;  Purposive sampling | Document information needs from the perspectives of Breast Cancer patients, family physicians and oncology specialist health care providers | None | 10 |
| [8] Maria Burton; 2015; UK | 33 | Age; Months since diagnosis; Treatment | Unreported | Semi-  structured interview | Framework analysis | Convenience sampling;  Purposive sampling | Information needs and preferences for this age group of women | None | 10 |
| [9] Belinda Thewes;  2003;  Australia | 24 | Age; Cancer stage; Months since diagnosis; Marital status; Number of children; Treatment; Menopausal status | Unreported | Focus group; individual semi-structured telephone interview | Standardised qualitative methodology (transcendental realism) | Convenience sampling;  Purposive sampling | Fertility-and menopause-related information needs of younger women with a diagnosis of early breast cancer | None | 10 |
| [10] Rana F. Obeidat; 2014; Jordan | 28 | Age; Education level; Marital status; Residence; Family history of breast cancer; Cancer stage at diagnosis; Surgery type; Treating facility | Jordanian | Semi-structured individual interview | Content analysis (conventional content analysis) | Convenience sampling;  Purposive sampling | Jordanian women's experiences of information exchange following diagnosis of early stage breast cancer | None | 10 |
| [11] Michelle M. Holmes; 2017; UK | 11 | Gender; Age; Race; Cancer diagnosis; Age of diagnosis; Time since end of active cancer treatment; Length of internet use; Frequency of internet use; Place of access to the internet | 91% white; 9% chose to not respond | Questionnaire; semi-structured telephone interview | Framework analysis | Convenience sampling;  Purposive sampling | Breast cancer survivors’ use of the internet when making decisions about CAM use | The theory of planned behaviour | 10 |
| [12] Kuang-Yi Wen; 2014; USA | 16 | Age; Education level; Household income; Time since diagnosis; Employment status; Marital status; Time lived in US; Cancer stage; Treatment | Chinese | Individual semi-structured interview | Content analysis ( qualitative content analysis) | Convenience sampling;  Purposive sampling | Experiences of Chinese American breast cancer survivors to better understand their information and  Communication needs | None | 10 |
| [13] Jane M. Ussher;  2013; Australia | 1965 | Gender; Age; Education level; Years since diagnosis; Menopausal status; Cancer stage at diagnosis; Marital status; Whether had children | White  (Anglo-Australian) | Online survey (closed and open ended  Questionnaire) | Thematic analysis | Convenience sampling;  Purposive sampling | Sexual information needs, experiences of obtaining such information, and patient communication | None | 10 |
| [14] Linda Rozmovits; 2004; UK | 28(15 breast cancer patients) | Age; Nationality; Network experience | Unreported | Focus group; Semi-structured interview | Thematic analysis | Convenience sampling;  Purposive sampling | Information needs of cancer patients, to solicit their views of Internet-based health information | None | 10 |
| [15] Marina Brown;2000; Australia | 6 | Cancer type; Treatment | Unreported | Semi-  Structured interview | Hermeneutics | Convenience sampling;  Purposive sampling | Experience of women who have had surgery for non-invasive breast cancer | None | 10 |
| [16] Sonia Kim Anh Nguyen;2013; USA | 56 | Age; Gender; Race; Cancer stage; Months since diagnosis | 77.8% White; 6.7% South Asian;15.5% unknown/  Other | Closed and open ended  questionnaire | Grounded theory | Convenience sampling;  Purposive sampling | Internet use by breast cancer patients highlighting search patterns and examining the impact of web-based information on the clinical encounter | None | 10 |
| [17] G.K.B. Halkett;  2010; Australia | 48 (34 breast cancer patients) | Treatment; Age; Marital status; Cancer stage | Unreported | Semi-  structured interview | Grounded theory | Convenience sampling;  Purposive sampling | Specific information needs of breast cancer patients who are receiving radiotherapy ；when patients prefer to receive specific information | The 1996 ‘Information Behaviour Model’ proposed by Wilson | 10 |
| [18] Nancy J. Burke  ;2016; USA | 38 | Age; Race; Primary language; Education level; Annual household income; Employment status; Insurance type; Age at diagnosis; Years since diagnosis; Cancer stage at diagnosis; Surgery type | 24% Asian; 13% African American/  Black; 18% Latino/  Hispanic; 18% Filipina; 24% White/  Caucasian; 3% mixed-race | Focus group | Thematic analysis | Convenience sampling;  Purposive sampling | Information needs and survivorship care plan preferences of low literacy, multi-lingual patients | None | 10 |
| [19] Emma Kemp;2018; Australia | 36 | Gender; Age; Cancer type; Cancer stage | Unreported | Semi-structured telephone interview | Thematic analysis | Convenience sampling;  Purposive sampling | (i) whether information and support-seeking preferences of women with advanced breast cancer (ABC) could be addressed in an online intervention, and (ii) how an existing intervention for patients with early stage cancer could be adapted for women with ABC. | None | 10 |
| [20] C. Kwok;  2014;  Australia | 23 | Age; Nationality; Length of stay in Australia; Marital status; Cancer stage; Diagnostic time; English proficiency; Family history of breast cancer; Years since diagnosis; Whether had children | Chinese-Australian | Focus group | Content analysis | Convenience sampling;  Purposive sampling | Perceptions of information needs and social support among Chinese-Australian breast cancer survivors | None | 10 |
| [21] Masoome Latifi;2018; Iran | 17 | Gender; Age; Surgery time; Marital status; Employment status; Economic status; Education level; Whether had children | Unreported | Semi-  structured interview | Content analysis  (qualitative content analysis) | Convenience sampling;  Purposive sampling | Information needs and information seeking motives of women with breast cancer after mastectomy | None | 10 |
| [22] Lynda G. Balneaves;  2016;  Canada | 39（22 breast cancer survivors） | Age; Study site; Marital status; Education level; Race; Household income; Cancer stage; Receptor status; Menopausal status; Treatment | 63.6% Caucasian; 18.2% Asian; 4.5% South Asian; 13.6% Missing | Semi-  structured interview | Thematic analysis | Convenience sampling;  Purposive sampling | Breast cancer survivors’ complementary therapies and general information and decision-making needs related to menopausal symptoms | None | 10 |
| [23] M. Elise Radina;  2011; UK | 35 | Age; Race; Education level; Marital status; Months since diagnosis; Cancer stage | White, Caucasian, or European American descent including Jewish, German descent, and first-generation Greek | Face-to-face semi-structured in-depth interview | Thematic analysis | Convenience sampling;  Purposive  sampling; Snowball sampling | Health information seeking behaviors (passive and active), use of health information, sources of health information, and how such information is or is not used in patients’ decision making about their treatment | A comprehensive and integrated model presented by Longo2005 | 10 |
| [24] Karen A Luker;  1996; UK | 105 | Age; Months since diagnosis; Marital status; Social class; Race | White British | Structured interview | Content analysis | Convenience sampling;  Purposive  sampling | Specific information needs and sources of information for 105 women with breast cancer at two time points | Case V of Thurstone's (1974) Law of Comparative Judgement | 10 |
| [25] B. Thewes;  2004; Australia | 18 | Age at diagnosis; age; Menopausal status; Cancer stage; Surgery type; Therapy; Months since adjuvant treatment; Marital status; Education level; Number and age of Children; Geographical location; Psychiatric history | Unreported | Formal semi-  structured telephone interview | Thematic analysis | Convenience sampling;  Purposive sampling | Identify the shared and unique needs of younger versus older survivors | None | 9 |
| [26] Lisa Wolf;2004; UK | 8 | Insurance type; Race; Age; Employment status; Treatment; Social class; Marital status; Months since surgery | Caucasian | Focus group | Framework analysis | Convenience sampling;  Purposive sampling | Experiences of women who had undergone breast reconstruction with a specific focus on their views on how they considered their information needs could best be met. | None | 10 |
| [27] Ross Gray;1997;  Canada | 24 | Age; Years since diagnosis; Social class; Education level | White | Semi-  structured interview | Thematic analysis | Convenience sampling;  Purposive sampling | (1) their personal experiences with breast cancer self-help group (2) the processes and structures of groups | Social learning theory | 10 |
| [28] Judy Gould;2006;  Canada | 65 | Age; Age at diagnosis; First language; Race; Marital status; Whether had children; Age of children; Treatment; Education level; Employment status; Household income; Insurance type | 72% Caucasian; 7% Canadian; 5% Jewish; 2% first nations; 4% French Canadian; 10% other | Focus group | Content analysis | Convenience sampling;  Purposive sampling | Experience of breast cancer for young women in Canada | None | 9 |
| [29] Lena Boman;1997; Sweden | 97 | Age | Unreported | Open ended questionnaire | Grounded theory | Convenience sampling;  Purposive sampling | Needs as expressed by a group of women after surgery for breast cancer in the setting of a short hospital stay | None | 9 |
| [30] Michelle Cappiello;  2007;  USA | 20 | Age; Race; Marital status; Education level; Treatment; Employment status; Living situation; Insurance type; Cancer stage | 90% Caucasian; 5% Hispanic; 5% Asian | Semi-  structured interview | Not clear | Convenience sampling;  Purposive sampling | Information and support needs of women following breast cancer treatment | None | 9 |
| [31] Lynda G. Balneaves  ;2007;  Canada | 20 | Age; Marital status; Education level; Individual annual income; Cultural identification; Treatment; Cancer stage | 10% Aboriginal Canadian; 55% Anglo-Saxon Canadian; 5% Chinese; 10% European; 15% Jewish; 5% South Asian | Semi-  structured interview | Grounded theory | Convenience sampling;  Purposive sampling | Personal and social processes women with breast cancer engaged in when making decisions about complementary and alternative medicine | None | 10 |
| [32] Sunmin Lee;2013;  USA | 9 | Age; Years since diagnosis; Cancer stage; Treatment status; Education level; Annual household income; Employment status; Marital status; Years lived in US; English proficiency; Cultural identification; Years of school in US | 55.6% very Asian; 22.2% Bicultural; 22.2% westernized | In-depth interviews | Thematic analysis | Convenience sampling;  Purposive sampling | Asian American women’s perceptions of quality of life and their breast cancer experiences | None | 10 |
| [33] Mary Ellen Shands;  2000;  USA | 19 | Age; Months since diagnosis; Education level; Age of children; Race; Household income; Surgery type; Cancer stage; Treatment | Caucasian | Semi-  structured interview | Content analysis | Convenience sampling;  Purposive sampling | Mothers' reported methods of interacting with school aged children about breast cancer | None | 9 |
| [34] Cannas Kwok;2011; Australia | 23 | Age; Months since diagnosis; Marital status; Primary language; Nationality; Whether had children; Family history of breast cancer; Years lived in Australia; Treatment; English proficiency | Chinese | Focus group | Content analysis | Convenience sampling;  Purposive sampling | Chinese-australian women’s perceptions of the meaning and experience of a breast cancer diagnosis,treatment and coping mechanism | None | 10 |
| [35] Laura D’Alimont;2012;  Canada | 12 | Age; Treatment; Cancer stage; Marital status; First language; Economic status; Chronic health conditions | Unreported | Focus group; One-on-one interview | Not clear | Convenience sampling;  Purposive sampling | To conduct a preliminary testing of the DA prototype to ensure that the format and information presented is clear and acceptable to patients | Ottawa Decision Support Framework | 9 |
| [36] Sunmin Lee;2012;  USA | 12 | Age; Years since diagnosis; Cancer stage; Treatment status; Education level; Annual household income; Employment status; Marital status; Years lived in US; English proficiency; Cultural identification; Years of school in US | 75% Chinese- or Korean American; 25% Asian American | In-depth interviews | Thematic analysis | Convenience sampling;  Purposive sampling | Patient physician communication processes in breast cancer care | None | 10 |
| [37] Jennifer Jing-Wen Wong;2011;  Canada | 16 | Age; Marital status; Chronic health conditions; Economic status; Cancer stage; Race | Caucasian | Focus group; One-on-one interview | Thematic analysis | Convenience sampling;  Purposive sampling | Information needs and unique illness experiences of older women with early stage breast cancer | None | 10 |
| [38]Christine R. Shaw;1994; USA | 11 | Gender; Age; Education level; Marital status | Unreported | Semi-  structured interview | Thematic analysis | Convenience sampling;  Purposive sampling | Information needs of women prior to breast biopsy | None | 10 |
| [39] Patricia Kenny;1999; Australia | 40 | Age; Cancer stage | Unreported | Semi-  structured interview | Content and thematic analyses | Convenience sampling;  Purposive sampling | The way women treated for early stage breast cancer perceived the treatment selection process. | None | 10 |
| [40] Jacqueline Barnes; 2000; UK | 32 | Age; Cancer stage; Social class; Marital status; Whether had children; Number and age of children; Occupation; Education level | Unreported | Semi-  structured interview | Content analysis | Convenience sampling;  Purposive sampling | Parents’ communication with their children about the diagnosis and initial treatment of breast cancer in the mother | None | 9 |
| [41]  G.K.B. Halkett;2007; Australia | 18 | Age; Marital status | Unreported | Face-to-face in-depth interview | Hermeneutic phenomenology | Convenience sampling;  Purposive sampling | The phenomenon of making decisions during the experience of early breast cancer；the types of decisions these patients are typically faced with | Heidegger’s (1996) philosophy | 10 |
| [42] Cathy Charles;  1998;  USA | 20 | Age; Treatment; therapy; Marital status; Occupation; Employment status; Cancer stage | Unreported | Open-ended, in-depth personal interview | Grounded theory | Convenience sampling;  Purposive sampling | (1) the extent to which women with early stage breast cancer perceived that they had treatment options; (2) their understanding of the risks and benefits associated with different treatment options; and (3) the role they wanted for  Themselves and for their oncologists in the treatment decision-making process. | None | 10 |
| [43] Linda L. Reaby;  1998; Australia | 95 | Age; Marital status; Race; Education level; Insurance type; Treatment; Cancer stage | 73% Australian- white | Semi-  structured interview | Thematic analysis | Convenience sampling;  Purposive sampling | The decision-making process used by women who had mastectomy as their surgical treatment for breast cancer | Decision-making model presented by Janis and Mann | 10 |
| [44] Lisa Beatty;2008; Australia | 34 (19 breast cancer patients) | Gender; Age; Months since diagnosis; Surgery type; Cancer stage; Current adjuvant treatment; Treatment status; Family history of breast cancer | Unreported | Focus group | Thematic analysis | Convenience sampling;  Purposive sampling | Concerns and needs of Australian women recently diagnosed with breast cancer | Bloom’s theory of psychosocial support | 10 |
| [45] Kirsten M. Weber;  2008;  USA | 33 weblogs ; 87 discussion board threads (N = 120) | Geographical location | Unreported | Network retrieval | Grounded theory | Convenience sampling;  Purposive sampling | The relational and communicative concerns confronting breast cancer patients or survivors and their family members | The relational turbulence model and the communication privacy management theory | 10 |
| [46] Carol L. McWilliama  ;2000; Canada | 11 | Gender; Age; Months since diagnosis; Marital status; Socio-economic class; Treatment; Geographical location; Race | Caucasian | In-depth interviews | Thematic analysis | Convenience sampling;  Purposive sampling | How women with breast cancer experience patient–physician communication to gain a greater understanding of effective approaches. | None | 9 |
| [47] Renee Royak-Schaler;  2008;  USA | 39 | Age; Income; Education level; Marital status; Age at diagnosis; Cancer stage at diagnosis; Treatment; Years since end of treatment; Therapy; Race | African American | Focus group | Thematic analysis | Convenience sampling;  Purposive sampling | Patient-physician communication from the patient’s perspective about guidelines and sources of information used in developing survivorship care and preferred avenues for information delivery to African American breast cancer survivors | None | 10 |
